# Genetic Factors Contributing to Interindividual Variability of α-Tocopherol Levels in Subcutaneous Adipose Tissue among Healthy Adult Males

**DOI:** 10.3390/nu16152556

**Published:** 2024-08-03

**Authors:** Mark Pretzel Zumaraga, Patrick Borel, Beatrice Gleize, Marion Nowicki, Djaffar Ould-Ali, Jean-François Landrier, Charles Desmarchelier

**Affiliations:** 1 Center for CardioVascular and Nutrition Research (C2VN), Aix Marseille Univ, INSERM, INRAE, 13005 Marseille, France; mark-pretzel.zumaraga@univ-amu.fr (M.P.Z.); patrick.borel@univ-amu.fr (P.B.); beatrice.gleize@inrae.fr (B.G.); marion.nowicki@geves.fr (M.N.); jean-francois.landrier@univ-amu.fr (J.-F.L.); 2 Department of Science and Technology, Food and Nutrition Research Institute, Bicutan, Taguig City 1631, Philippines; 3 Plastic & Anesthetic Surgery Department, Clinique Internationale du Parc Monceau, 75017 Paris, France; djaffaroa@gmail.com; 4 Institut Universitaire de France (IUF), 75000 Paris, France

**Keywords:** vitamin E, genetic variants, adipocyte, antioxidant

## Abstract

In humans, α-tocopherol (α-TOC) is mainly stored in adipose tissue, where it participates in preventing damages induced by inflammation and reactive oxygen species. Factors, including genetic ones, that explain adipose tissue α-TOC concentration remain poorly understood. This study, therefore, aimed to characterize the interindividual variability of adipose tissue α-TOC concentration in healthy individuals and to identify single nucleotide polymorphisms (SNPs) associated with it. The study used a randomized cross-over design with 42 healthy adult males. α-TOC concentration was measured in fasting plasma and periumbilical adipose tissue samples, both at fast and 8 h after consumption of three standard meals. Partial least squares (PLS) regression was performed to identify SNPs associated with the interindividual variability of adipose tissue α-TOC concentration. Adipose tissue α-TOC concentration was not associated with fasting plasma concentration (Pearson’s *r* = 0.24, 95% CI: [−0.08, 0.51]). There was a high interindividual variability of adipose tissue α-TOC concentration (CV = 61%). A PLS regression model comprising 10 SNPs in five genes (*PPARG*, *ABCA1*, *BUD13*, *CD36*, and *MGLL*) explained 60% (adjusted *R*^2^) of the variability of this concentration. The interindividual variability of adipose tissue α-TOC concentration in humans is due, at least partly, to SNPs in genes involved in α-TOC and triglyceride metabolism.

## 1. Introduction

Vitamin E (VE) is a generic term that usually describes a group of eight naturally occurring lipid-soluble molecules: four tocopherols (TOC) (α, β, γ, and δ) and four tocotrienols (α, β, γ, and δ) [1]. Based on their capacity to effectively prevent human VE deficiency disease, it has recently been suggested to restrict the attribute of VE either only to the α-TOC stereoisomer in the *RRR* conformation [2], which is of natural origin, or to the four stereoisomers of all *rac*-α-TOC, which is of synthetic origin, in the 2*R* conformation [3]. α-TOC possesses well-established chain-breaking antioxidant properties, especially against peroxyl radicals [1,4,5]. Nevertheless, it also possesses non-antioxidant properties [6]. For example, it activates multiple enzymatic activities involved in inflammatory and allergic reactions [7,8], cell proliferation [9], platelet aggregation [10], and bone mass regulation [11], while its phosphorylated metabolite has been shown to modulate gene expression, exhibiting hormone-like properties [12,13].

In humans, as much as 90% of total body VE is stored in white adipose tissue, particularly in the lipid droplets of adipocytes [14]. This VE pool consists of about two-thirds α-TOC and one-third γ-TOC, although this ratio can vary depending on the dietary habits of the population [15,16,17,18]. VE in adipose tissue has two main origins: it is taken up from lipoproteins of intestinal origin, i.e., chylomicrons but also HDL, following VE consumption; and it is taken up from lipoproteins of hepatic origin, i.e., VLDL and HDL [19]. The exact biological roles of α-TOC in adipose tissue are not yet fully elucidated, and significant knowledge gaps still remain, particularly in humans [20]. Some animal studies have shown that α-TOC supplementation resulted in decreased adipose tissue fibrosis [21], oxidative stress [22], inflammation [23], and increased circulating adiponectin concentration [22,24] in obesity. These modifications are at least partly due to the effects of α-TOC on gene expression [25,26], with the notable involvement of PPARγ [24,25,27], a key nuclear receptor in the regulation of adipocyte biology. 

Several studies have shown that α-TOC concentration in adipose tissue exhibits a high interindividual variability (Table 1), with a CV of approximately 50%. Schäfer and Overvad reported a high correlation (*r* = 0.76, *n* = 20) between adipose tissue total tocopherol concentration and dietary VE intake [28]. Together with the fact that, in contrast to circulating α-TOC concentration, α-TOC concentration in adipose tissue remains stable over time [18], this prompted them to suggest its use as a marker to assess regular VE intake. However, other authors did not observe such a high correlation [16,17,29]. For example, in a large study involving 458 healthy participants in Costa Rica, El-Sohemy et al. reported a poor correlation, i.e., *r* = 0.15, between concentration in the subcutaneous adipose tissue and dietary α-TOC intake. Therefore, factors other than dietary α-TOC intake alone explain this high interindividual variability. The absorption, transport, metabolism, and elimination of α-TOC in adipose tissue are governed by numerous proteins, most of which are common to the metabolic pathways of other lipophilic compounds [30,31,32]. The involvement of proteins suggests that polymorphisms in their encoding genes could influence α-TOC concentration in adipose tissue by modulating their expression or activity [33]. In support of this, several single nucleotide polymorphisms (SNPs) have been previously associated with fasting plasma α-TOC concentration [33,34,35,36,37,38], and we have also shown that a combination of 28 SNPs was associated with α-TOC bioavailability [39]. To date, there is no study on the association between genetic variants and α-TOC concentration in adipose tissue. Hence, this study aimed to characterize the interindividual variability of α-TOC concentration in the subcutaneous adipose tissue of healthy participants and to identify SNPs associated with it, following a candidate gene approach.

## 2. Materials and Methods

### 2.1. Study Design 

This study is an ancillary study of a randomized cross-over clinical trial (ClinicalTrials.gov registration number: NCT02100774, dated 27 March 2014) carried out between January 2009 and July 2010. Its aim was to identify genetic variants associated with the interindividual variability of the postprandial bioavailability of lipid micronutrients, i.e., α-TOC, β-carotene, cholecalciferol, lutein, and lycopene [39,40,41,42]. There are no existing data regarding the association between genetic variants and α-TOC concentration in adipose tissue. Hence, no power calculation could be carried out. Briefly, 42 healthy, non-overweight, non-obese (BMI < 25 kg/m^2^) and non-smoking adult males, whose baseline characteristics are shown in Table 2, consumed three test meals (as described elsewhere). The first test meal had no added micronutrients and was called the control meal. The second test meal included a capsule containing *RRR*-α-tocopheryl acetate equivalent to 67 mg (100 IU) α-TOC (Holland & Barrett, Nuneaton, Warwickshire, England) and was called the α-TOC meal. The third test meal had 100 g tomato puree as a source of lycopene [42] and other carotenoids, i.e., β-carotene and phytofluene [40,43], and was called the tomato meal. The quantities of α-TOC present in the control, the tomato, and the α-TOC meal were 7.9, 10.2, and 74.9 mg, respectively, as calculated based on food composition tables (available at: https://ciqual.anses.fr/, accessed on 31 October 2023) and on the capsule composition. Participants were advised to limit their intake of α-TOC-rich foods 2 days prior to each test meal (a food list was given to the participants). A day before each test meal, participants were advised to take dinner between 7 and 8 p.m., without intake of any type of alcoholic beverages. They were also asked to abstain from consuming any food or beverage other than water after dinner and until they came to the Center for Clinical Investigation (la Conception Hospital, Marseille, France). On the day of the clinical trial, participants were given instructions to consume the test meal within 20 min at a relatively uniform pace. After every test meal consumption, they were asked to refrain from consuming any other food for the next 8 h, except water provided with the meal. Subcutaneous adipose tissue samples from the periumbilical region were obtained from study participants by a trained physician at 2 time points: at fast and 8 h after the intake of the 3 test meals. Briefly, after local anesthesia with xylocaine (2%), adipose tissue was aspirated using a 10 mL Luer Lock syringe, through a 15 Gauge cannula (15 cm in length and 1.5 mm internal diameter). Typically, the aspirate contained a quantity ranging from 100 to 200 mg of subcutaneous adipose tissue. The collected adipose tissue was promptly transferred into an ice-water bath and covered with aluminum foil until extraction. The study was approved by the Regional Committee on Human Experimentation on 8 October 2008 (N 2008-A01354-51, Comité de Protection des Personnes Sud Méditerranée I, Marseille, France). The steps taken were in accordance with the 1983 revision of the 1975 Declaration of Helsinki. Written informed consent was acquired from each participant involved.

### 2.2. Biochemical Measurements

Fasting plasma concentrations of total cholesterol (TC), HDL-C, LDL-C, triglyceride, glucose, and hemoglobin were measured as previously described [44].

### 2.3. Plasma and Subcutaneous Adipose Tissue α-TOC Concentration Measurements

Fasting plasma samples and postprandial chylomicron samples for the quantification of α-TOC were processed as previously described [39]. For the quantification of α-TOC in adipose tissue samples, around 50 mg (fresh weight) adipose tissue was crushed in 300 µL of phosphate buffered saline solution with two 3-mm diameter stainless steel balls using an MM301 ball mill (Retsch, Eragny-sur-Oise, France). Then, a 50 µL aliquot was collected for protein quantification by the BiCinchoninic acid Assay kit (Pierce, Montluçon, France) after dilution to 1/5 in phosphate-buffered saline solution. Lipids, including α-TOC, were extracted from the remaining 250 μL using 2 mL trichloromethane/methanol (1/1, *v*/*v*) and 0.9 mL phosphate buffered saline solution. All extractions were performed at room temperature under yellow light to minimize light-induced damage. The dried extract was incubated at 37 °C for 1 h 30 min with 100 μL of an ethanolic pyrogallol solution (12%, *w*/*v*) and 1 mL of an ethanolic potassium hydroxide solution (5.5%, *w*/*v*). After incubation, the sample was cooled to room temperature, and tocopheryl nicotinate (>95% purity) was added as an internal standard. The mixture was extracted twice by using 3 mL of hexane. The extract was evaporated to dryness under nitrogen and then solubilized in 100 μL methanol/dichloromethane (65/35, *v*/*v*) for downstream HPLC analysis. The HPLC apparatus consisted of a separation module (P680 HPLC Pump and ASI-100 Automated Sample Injector, Dionex SA, Villebon sur Yvette, France) and a UVD340U photodiode array detector (Dionex SA). Separation was achieved using a 10 mm × 4.0 mm Modulo-Cart guard column, with a 2 μm particle size (Interchim, Montluçon, France) followed by a 250 mm × 4.6 mm, 5-μm particle size C18 Zorbax Uptisphere column (Interchim). The isocratic mobile phase was composed of 100% HPLC-grade methanol (Carlo Erba–SDS, Peypin, France) maintained at 35 °C with a flow rate of 1.5 mL/min. α-TOC was detected at 290 nm and identified via spectra, and a retention time coincident with authentic standard (Sigma-Aldrich, Saint Quentin Fallavier, France). Peaks were integrated using Chromeleon software (version 6.80, Dionex SA). Quantitation was performed using external calibration curves normalized to internal standards as previously described [39].

### 2.4. Saliva DNA Extraction and Genotyping

Saliva samples were processed using the Oragene kit (DNA Genotek, Ottawa, ON, Canada) to extract approximately 25 μg of genomic DNA. Whole-genome genotyping was carried out using HumanOmniExpress BeadChips (7.13 × 10^5^ SNPs per chip; Illumina, San Diego, CA, USA), as previously described [43].

### 2.5. Candidate Gene and SNP Selection 

Following a literature review, a total of 39 candidate genes were selected (Supplementary Table S1 and Figure S1). The selection was made based on their association, whether previously demonstrated or putative, with circulating or adipose tissue α-TOC concentration. This association could be direct, through their role in adipocyte α-TOC uptake or metabolism, or indirect, through their influence on blood α-TOC concentration or adipocyte lipid droplet metabolism. Of the corresponding 2453 SNPs on the DNA chips, we first excluded SNPs for which the genotype call rate was <95% and SNPs that presented a significant departure from the Hardy–Weinberg equilibrium (*p* < 0.05; chi-squared test) (490 SNPs excluded), leaving 1963 SNPs. Then, for each candidate gene, we excluded SNPs in high linkage disequilibrium (LD, *R*^2^ > 0.80) and kept the tag SNPs as identified by LD TAG SNP Selection tool from the SNPinfo Web Server (HapMap, European, i.e., CEU, population, accessible at https://snpinfo.niehs.nih.gov, accessed on 12 January 2023) (623 SNPs excluded), leaving 1340 SNPs. Note that further analysis was performed on SNPs not available in the SNPinfo Web Server database: when 2 SNPs were perfectly correlated (i.e., *R*^2^ = 1.0), one was randomly kept, leading to the exclusion of an additional 121 SNPs. The 1219 remaining SNPs were tested under both additive and dominant models. SNPs with fewer than 5 observations in a genotypic group were excluded from further analysis, leaving 359 and 1034 SNPs in the additive and dominant models, respectively.

### 2.6. SNP Function Prediction

The functional consequences of both the intronic and regulatory variants were predicted by the online tool RegulomeDB (available at https://regulomedb.org/, accessed on 1 March 2023). The RegulomeDB probability score spans from 0 to 1, where a score of 1 suggests a higher likelihood that the variant has a regulatory role [45].

### 2.7. Statistical Analysis

Data were expressed as mean ± SEM. Pearson’s *r* was expressed with 95% CI. Adipose tissue α-TOC concentrations measured at fast and 8 h after consumption of the 3 test meals were analyzed with linear mixed models (LMM), using a full factorial design with meal (control, α-TOC, and tomato puree) and time (fasting and 8 h post-meal) as fixed within-subject variables and participant as the random variable. Five different covariance structures were tested, i.e., autoregressive order one, diagonal, scaled identity, unstructured covariance, and compound symmetry, using Akaike’s Information Criterion (AIC). Residual scatterplots were examined to determine departure from homoscedasticity, while QQ plots were used to determine departure from normality. Since neither meal nor time had a significant effect on α-TOC concentrations in the adipose tissue, which was also verified by paired *t*-tests comparing each meal adipose tissue α-TOC concentrations at baseline vs. post-meal (see Section 3 for detailed explanation), we decided to treat all α-TOC concentration measurements within each participant as technical replicates. The presence of outliers among the technical replicates was assessed using 2-tailed Grubbs’ tests (available at https://www.graphpad.com//quickcalcs/Grubbs1.cfm, accessed on 7 December 2022). Subsequently, the arithmetic mean of α-TOC concentration in adipose tissue for each participant was calculated and is hereafter simply referred to as α-TOC concentration in the adipose tissue. The bilinear relationship between α-TOC concentration in the adipose tissue and anthropometric measurements and plasma concentration of other lipids was measured using Pearson’s *r*. CV of plasma and adipose tissue α-TOC concentrations were compared according to Forkman [46]. For all tests, the bilateral alpha risk was *α* = 0.05. Statistical analyses were performed using SPSS 28 (SPSS Inc., Chicago, IL, USA).

In order to identify the combination of SNPs explaining best the variance in adipose tissue α-TOC concentration, we followed a 2-step approach, combining dimension reduction by univariate filtering followed by partial least squares (PLS) regression, as previously applied with SNPs [43,47,48]. The univariate filtering step involved selecting SNPs with a *p*-value < 0.05 (Wald test asymptotic *p*-value) using PLINK (v1.07, http://pngu.mgh.harvard.edu/purcell/plink/, accessed on 12 January 2023). Additionally, covariates (i.e., age, BMI, fasting lipid concentrations, etc.) were also selected if they exhibited a correlation coefficient with the adipose α-TOC concentration that was significantly different from zero, according to 95% CI. A PLS regression model including all thus selected variables, i.e., 78 (77 SNPs and fasting plasma cholesterol concentration), coded in units of variance, was then built. Variables were ranked according to their variable importance in the projection (VIP) value, which estimates the contribution of each variable in the projection used in the PLS regression model, and several PLS regression models were then generated using increasing VIP threshold values as described in detail elsewhere [44,49]. The model maximizing the adjusted *R*^2^ (Equation (1)) and having a significant *p*-value after cross-validation ANOVA [50] was selected.

(1) $$Adjusted\;R^{2}=1-\frac{\left(1-R²)(n-1\right)}{n-k-1}$$

with *n* the sample size and *k* the number of variables in the model (excluding the constant). Robustness and stability of the selected model were validated by leave-*k*-out cross-validation [51], regression coefficient stability testing [43], and permutations, where *R*^2^ and cross-validated *R*^2^ were tested after 100 random permutations of the Y variable, i.e., adipose tissue α-TOC concentration [52] (see additional validations of the PLS regression model in Supplementary Materials). SIMCA^®^ Multivariate Data Analytics Solution software (Version 17.0.0.24543, Umetrics, Umeå, Sweden) was used for all multivariate data analyses, robustness, and stability tests. 

### 2.8. Retrospective Multivariate Power Analysis Calculations

A retrospective sample size power calculation was conducted using the online tool MetaboAnalyst 6.0 (accessible at https://new.metaboanalyst.ca, accessed on 5 June 2024). By utilizing the Power Analysis feature of MetaboAnalyst 6.0, we determined the minimum sample size required to achieve statistical significance for a dataset consisting of the combination of SNPs identified through PLS regression analysis. To facilitate this analysis, the sample was divided into two groups based on the median adipose tissue *α*-TOC concentration (median = 109.9 nmol/g protein). 

## 3. Results

### 3.1. α-TOC Concentration in the Adipose Tissue

**Figure 1:**
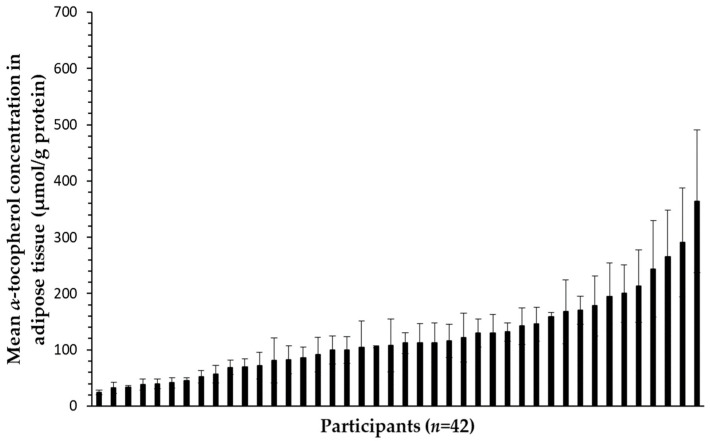
Adipose tissue α-TOC concentration of the study participants. α-TOC concentration in adipose tissue (nmol/g protein) of each participant was calculated as the mean of the concentrations measured in periumbilical samples collected on three occasions, at fast and 8 h after consumption of three test meals, separated by a washout period of at least three weeks. Values are means (*n* = 2 to 6) with their SEM. Participants (*n* = 42) were sorted by increasing α-TOC concentration in adipose tissue.

Neither the sampling time (*p* = 0.935), i.e., fasting or 8 h after the test meal, nor the test meal ingested (*p* = 0.734), i.e., control, α-TOC and tomato puree, had a significant effect on adipose tissue α-TOC concentration (also verified by paired *t*-tests; see Supplementary Table S3). Therefore, all adipose tissue α-TOC concentration measurements within each participant were treated as technical replicates, and the mean adipose tissue α-TOC concentration of each participant was then calculated, i.e., most of the time, the mean of six values (Figure 1). The concentration of α-TOC in the adipose tissue exhibited a high interindividual variability, with a CV of 61%. This was significantly higher (*p* < 1.0 × 10^−5^) than the interindividual variability of fasting plasma α-TOC concentration, which had a CV of 25%.

There was no significant correlation between adipose tissue α-TOC concentration and age, BMI, fasting plasma α-TOC concentration, or postprandial chylomicron α-TOC concentration. We found a significant correlation between adipose tissue α-TOC concentration and total cholesterol concentration but not with LDL-C, HDL-C, or triglyceride concentration (Table 3).

### 3.2. SNPs Associated with the Interindividual Variability of α-TOC Concentration in the Adipose Tissue

We first measured the association between individual SNPs and adipose tissue α-TOC concentration (Table 4). Eighteen SNPs exhibited a significant *p*-value under the additive model and 59 under the dominant model. The distribution of SNPs locations was as follows: 48% intergenic, 47% intronic, and 5% near the 3′ or 5′ untranslated region. Unstandardized regression coefficients (*B* coefficients), which represent the mean change in adipose tissue α-TOC concentration for each additional copy of the minor allele under the additive model and in the presence of the minor allele under the dominant model, are provided in Table 4. Note that many SNPs had high RegulomeDB scores, i.e., close to 1, which indicates a good probability that they are located within a regulatory region and could, therefore, influence gene expression. 

### 3.3. Combinations of SNPs Associated with the Interindividual Variability of α-TOC Concentration in the Adipose Tissue

We used PLS regression to find the best combination of SNPs and covariates to explain the interindividual variability of adipose tissue α-TOC concentration. As shown in Supplementary Table S2, the model including all thus selected variables (77 SNPs and fasting total cholesterol concentration) could explain a high part of the variance in this phenotype (*R*^2^ = 0.80), but this estimation was positively biased due to the high number of predictors included in the model. Therefore, to improve the model and find a combination of SNPs more predictive of this phenotype, we sequentially filtered out SNPs and covariates that made a less important contribution, i.e., those that displayed the lowest VIP values. With the application of several VIP value thresholds, we selected a model that included 12 SNPs, of which 10 were not in LD (Supplementary Table S5). The 10 SNPs were located in or near five genes (Table 5) and explained 60% of the variance (adjusted *R*^2^). The model was first validated by cross-validation ANOVA (*p* = 2.7 × 10^−9^) (Supplementary Table S2). Then, its robustness and stability were validated by three additional methods (i.e., leave-*k*-out cross-validation, regression coefficient stability test, and random permutations) (Supplementary Table S4 and Figures S2 and S3). The retrospective multivariate power analysis evaluation indicated that a sample size of at least 16 participants per group was needed to achieve a statistical power of 85% at a false discovery rate (FDR) adjusted *p*-value of 0.001 (Supplementary Figure S4). This confirms that the sample size used in this study was adequate.

### 3.4. Genetic Score of α-TOC Concentration in the Adipose Tissue

With the knowledge of a participant’s genotype at the 10 SNPs in the selected model, it was possible to calculate his adipose tissue α-TOC concentration using the following equation (adjusted *R*^2^ = 0.60):

(2) $$Adipose\;tissue\;\alpha -TOC\;concentration=35.7+\sum_{i=1}^{10}\left(r_{i}\right)\times number\;of\;minor\;allele\;{SNP}_{i}$$

with *r_i_* the unstandardized regression coefficient of the *i*th SNP in the PLS regression model (provided in Table 5). When SNPs were entered under the dominant model, participants homozygous for the lesser frequent allele were grouped with heterozygous participants and the number of minor alleles for both these groups was considered to be 1.

## 4. Discussion

The primary goal of this study was to identify SNPs and other covariates associated with the interindividual variability of α-TOC concentration in adipose tissue, the major VE storage site in the human body. While the concentration of α-TOC in plasma can undergo significant modifications within a few days following dietary changes [15], its concentration in adipose tissue changes at a much slower rate, i.e., over several weeks to a few years, except in pathological situations, e.g., burn patients [53]. For example, Handelman et al. provided four healthy adult males 800 mg all *rac*-α-TOC per day, which, respectively, exceeds the current French and US recommended dietary allowance by 40 and 27 times, for a duration of one year. The authors showed that only one participant exhibited a significant increase in α-TOC concentration in adipose tissue. Moreover, after discontinuation of supplementation for one year, no decrease was observed [18]. In the present randomized cross-over clinical trial, participants received three meals containing 7.9 mg, 10.2 mg, and 74.9 mg of α-TOC, separated by a washout period of at least 3 weeks. Biopsy samples were collected both in the fasting state and 8 h after meal intake. Therefore, we did not anticipate any significant change in α-TOC concentration in the adipose tissue. This was confirmed by the observation that neither the biopsy time nor the ingested test meal had a significant effect on α-TOC concentration in the adipose tissue. Consequently, all α-TOC concentration measurements in a participant’s adipose tissue were treated as technical replicates to obtain a more accurate estimate.

In this group of healthy adult males, α-TOC concentration in the adipose tissue exhibited a high interindividual variability, with a CV of 61%. This is fairly high, considering participants formed a relatively homogenous group, suggesting that it may be even higher in the general population. Nonetheless, this value falls within the range of previously reported variabilities (Table 1) [16,17,28,29]. To better characterize this variability, we first measured the association of α-TOC concentration in the adipose tissue with participants’ age, BMI, and blood lipid concentrations. Importantly, α-TOC concentration in the adipose tissue was not significantly correlated with fasting plasma α-TOC concentration (*r* = 0.24 [95% CI: −0.07, 0.51]). Varying correlations between α-TOC concentration in adipose tissue and plasma have been previously reported, i.e., 0.34 (deattenuated Pearson’s *r* in 77 males and females) [29], 0.51 (deattenuated Pearson’s *r* in 90 males and 120 females) [17], and 0.27 (Spearman’s *ρ* in 482 males and females) [16]. However, they remain relatively low, which is partly due to the regulation of circulating α-TOC concentration under the control of the liver [15,19]. It is also in support of our hypothesis, i.e., α-TOC concentration in adipose tissue is not only influenced by circulating α-TOC concentration but rather depends on several processes, such as α-TOC distribution in blood lipoproteins, α-TOC uptake by adipocytes, α-TOC metabolism in adipocytes [54,55]. Since these processes involve several proteins, and thus genes, we investigated the association of SNPs in these genes with the interindividual variability of α-TOC concentration in the adipose tissue, and we here report a combination of 10 SNPs in 5 genes significantly associated with it. These associations are discussed below. 

Adipose tissue α-TOC concentration was associated with rs3211958 in *CD36*, which is a scavenger receptor with a high affinity to a variety of ligands, including lipoproteins (i.e., VLDL, oxidized LDL) carrying various lipids and fat-soluble micronutrients, such as α-TOC, as demonstrated in both human and mouse studies [56,57,58,59]. In the bloodstream, α-TOC is almost exclusively carried by lipoproteins. Because CD36 recognizes a broad variety of lipid ligands, we hypothesize that recognition of α-TOC by CD36 is plausible. Similar to SR-BI (scavenger receptor class B type 1), CD36 could serve as a docking site, thereby participating, either directly or indirectly, in the adipocyte uptake of circulating lipid molecules, including α-TOC. Therefore, the observed association between rs3211958 and α-TOC concentration in the adipose tissue suggests the involvement of the encoded CD36 protein in influencing α-TOC internalization by adipocytes. Furthermore, rs3211958 exhibits moderate linkage disequilibrium (LD) (*R*^2^ = 0.59) with rs1527479 in the same gene. The latter has been linked to plasma α-TOC concentration in a study involving 993 participants from 10 European countries, underscoring the potential functional importance of rs3211958 [34].

We have also identified an association between α-TOC concentration in the adipose tissue and rs3773161, located in *MGLL. MGLL* encodes for monoglyceride lipase, which participates in the mobilization of adipose tissue triglyceride stores by hydrolyzing monoglycerides to free glycerol and a fatty acid [60]. As most α-TOC in adipocytes is stored in the bulk lipid droplet [14], this SNP could impact α-TOC concentration in adipose tissue by influencing the amount of triglycerides available to store α-TOC. Moreover, the activity of this enzyme produces free fatty acids, which are more prone to oxidation than esterified fatty acids. Since α-TOC acts as an antioxidant, the production of free fatty acids in the adipocyte by MGLL could influence the catabolism of α-TOC and, therefore, its concentration.

Two SNPs (rs709158 and rs13076933) in *PPARG* were associated with α-TOC concentration in the adipose tissue. *PPARG* encodes for the peroxisome proliferator-activated receptor, which is an orphan member of the nuclear hormone receptor superfamily that acts as both a transcription factor and a lipid sensor. *PPARG* is expressed at particularly high levels in adipose tissue, where it regulates adiposity by controlling adipocyte differentiation and fat metabolism [61] through the modulation of many genes and metabolic pathways [27]. Landrier et al. showed that incubation of cultured adipocytes with α-TOC and α-TOC supplementation in mice led to an increased relative expression of *PPARG* [24], which was due to the increased intracellular production of 15d prostaglandin J2, which is a natural PPARG ligand [27,62]. We can speculate, from a molecular point of view, that polymorphisms in *PPARG* may disturb the increase in its transcriptional activity due to α-TOC, which could be responsible, at least in part, for downstream modulation of many genes and metabolic pathways, i.e., adiponectin induction, lipid droplet formation, among others [36]. 

Out of the 10 SNPs in the final PLS regression model, 4 were located in *ABCA1* (rs1561166, rs12686004, rs2275542, and rs4743764), which encodes for ATP-binding cassette transporter A1. ABCA1 mediates the efflux of lipids to apoA1 and small HDL particles, including that of α-TOC by enterocytes [63], macrophages [31], and hepatocytes [64]. *ABCA1* has been shown to be also expressed and functional in adipocytes [65]. Indeed, the adipose tissue has been shown to be able to efflux significant quantities of cholesterol to HDL particles, both in vitro and in vivo [66]. Therefore, our findings are in support of the secretion by adipose tissue of a fraction of α-TOC in HDL, mediated by ABCA1. 

Two SNPs, rs1783225 and rs1648364, in *BUD13*, were associated with α-TOC concentration in the adipose tissue. *BUD13* encodes for BUD13 homolog protein, which is one of the subunits of the retention and splicing complex, previously identified in yeast as a splicing factor that affects nuclear pre-mRNA retention [67]. SNPs in *BUD13* have been previously associated with circulating α-TOC concentrations in genome-wide association studies in European and Asian populations [33,35,68]. Although no direct mechanism linking BUD13 and α-TOC metabolism has been identified, a possible explanation might lie in the fact that *BUD13* is located in the same gene cluster on chromosome 11 as *APOA1/C3/A4/A5*. These genes encode key proteins involved in lipoprotein metabolism, and additionally, a SNP in *APOA5* has been associated with circulating α-TOC concentration in a genome-wide association study [69]. 

Of note, three SNPs in or near *TTPA* were found to be associated with adipose tissue α-TOC concentration following univariate analysis (Table 4), although none was retained in the final PLS model. *TTPA* encodes for α-TOC transfer protein, a soluble protein mostly expressed in the liver but that is also found in adipose tissue [70]. It binds α-TOC with high selectivity and affinity, and it is involved in its trafficking to cell membranes before its uptake by nascent lipoproteins, at least in the liver [3]. Of these 3 SNPs, rs6472073 has been previously found to be associated with the serum response to VE supplementation in 2112 middle-aged male smokers in a genome-wide association study, although with marginal significance [35]. 

This study has limitations. α-TOC concentration in adipose tissue is a complex phenotype that results from the uptake of circulating α-TOC and its metabolism in this tissue [71,72]. Many genes are consequently involved, and thus, a thorough investigation of the association between genetic variants and this phenotype should ideally include all genes involved in this phenotype. Since we followed a candidate gene approach, important genes whose association with this phenotype is not yet known may have been left out of the analysis. Additionally, several SNPs in the selected candidate genes were not entered in the PLS regression analysis because either they were not expressed on the BeadChips or they were excluded from the analysis (as explained in Methods). Moreover, considering the sample size, this study should be regarded as exploratory, and since only Caucasian males were investigated, the associations reported herein need to be validated externally in females as well as in other ethnic groups with a larger sample size. Indeed, females have been reported to exhibit higher adipose tissue (and plasma) α-TOC concentration compared to males, suggesting a different α-TOC metabolism, possibly due to differences in the expression of the proteins involved. Furthermore, genotyping of different ethnic groups has shown that genetic variants are not linked similarly, meaning haplotype blocks are not the same across different populations. Thus, it is possible that a genetic variant in LD with a nearby SNP that affects α-TOC concentration in one population may not be linked to that causal SNP in another population, leading to variable results in association studies. Moreover, SNPs involved in the interindividual variability of adipose tissue α-TOC concentration might exhibit significantly different allele frequencies depending on the ethnic group investigated. Finally, α-TOC concentration was measured only in periumbilical, i.e., subcutaneous, adipose tissue, and we can not exclude that the associations we report herein differ in other adipose tissue, e.g., visceral adipose tissues. On the other hand, one of the strengths of the study was that the phenotype analyzed, i.e., α-TOC concentration in adipose tissue, was calculated as the average from up to six measurements, thereby providing a more accurate estimate.

## 5. Conclusions

**Figure 2:**
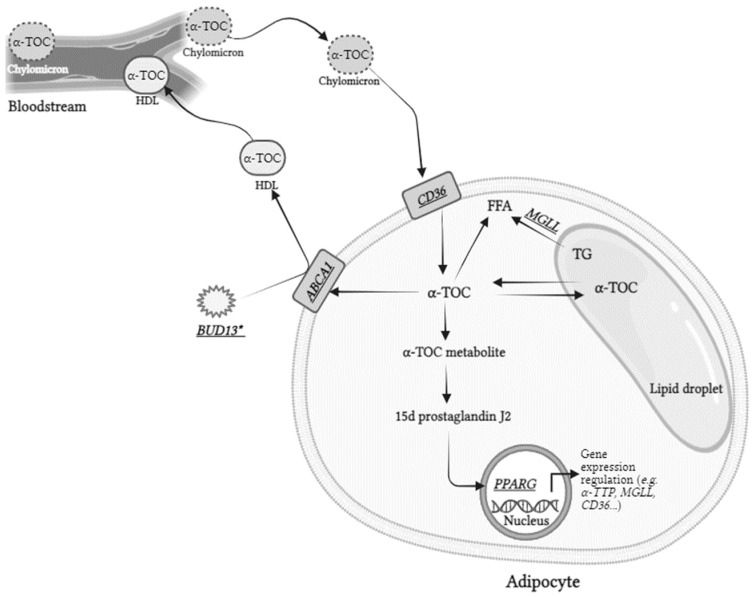
Genes whose SNPs were associated with the interindividual variability of adipose tissue α-TOC concentration. Genes displayed are those whose SNPs were in the partial least squares regression model, explaining best the variability of adipose tissue α-TOC concentration. This model included 10 SNPs in 5 genes. * As *BUD13* is not known to have an effect on α-TOC metabolism, these associations could be due to the fact that SNPs in or near this gene are in LD with SNPs in *APOA1/C3/A4/A5*, which is a key gene cluster in lipoprotein metabolism and α-TOC blood clearance (see Section 4). The full names of the genes are shown in Supplementary Table S1: TG—triglycerides; FFA—free fatty acid; HDL—high-density lipoprotein.

Taken together, this study allowed us, for the first time, to identify genes and SNPs that are associated with α-TOC concentration in human adipose tissue (schematized in Figure 2). Nevertheless, these results can serve as a first basis for future studies on the identification of the genetic variants that modulate α-TOC concentration in this tissue, with the ultimate goal to help identify individuals at risk of low α-TOC concentrations in adipose tissue, the body’s primary α-TOC storage site. Future studies should ideally include a more diverse sample population, particularly with regard to sex and ethnic background, and explore other (epi)genetic variants, i.e., other SNPs but also copy number variants or epigenetic modifications. As suggested previously, future clinical studies examining the impact of VE supplementation on adipose tissue biology should consider participants’ genetic characteristics for better interpretation of results [16,73]. Consequently, we anticipate that these initial findings will prompt further research to deepen our understanding of how α-TOC is metabolized in adipose tissue, an organ crucial for the regulation of energy metabolism and endocrine functions, whose impairments are associated with cardiovascular disease, diabetes, obesity, and certain cancers [20,33,74].

## Figures and Tables

**Table 1:** Interindividual variability of adipose tissue α-TOC concentration in selected observational studies.

Author/s	Publication Year	*n*, Population	Mean	SD	%CV ^a^	Ref.
Schäefer and Overvad,	1990	20 healthy participants from Denmark	409.8 mmol/mol triglyceride ^b^	206.7	50.4	[28]
Kardinaal et al.	1995	85 healthy participants from the Netherlands (47 females and 38 males)	281.0 μg/g total fatty acid ^c^	152.0	54.1	[29]
			240.0 μg/ g total fatty acid ^d^	106.0	44.2	
Su et al.	1998	213 healthy participants from Spain, Germany, the Netherlands, Northern Ireland and Switzerland (122 females and 91 males)	355.2 μg/g total fatty acid ^c^	181.5	51.1	[17]
			268.4 μg/g total fatty acid ^d^	147.3	54.9	
El-Sohemy et al.	2001	458 healthy participants from Costa Rica (111 females and 347 males)	123.1 μg/g adipose tissue ^c^	69.5	56.5	[16]
			82.9 μg/g adipose tissue ^d^	55.9	67.4	

^a^ The value of %CV was calculated when SD or SEM was reported. ^b^ The value provided included participants of both sexes. ^c^ The indicated value pertains solely to female participants. ^d^ The indicated value pertains solely to male participants.

**Table 2:** Baseline characteristics of the study participants (*n* = 42).

Characteristic	Mean (SEM)
Age, y	31.3 (1.9)
Weight, kg	73.6 (1.3)
BMI, kg/m^2^	23.0 (0.3)
Total cholesterol, g/L ^a^	1.6 (0.1)
Triglycerides, g/L ^a^	0.8 (0.1)
HDL-C, g/L ^a^	0.5 (0.0)
LDL-C, g/L ^a^	1.0 (0.1)
Glucose, mmol/L ^a^	4.7 (0.1)
Hemoglobin, g/dL ^a^	15.0 (0.1)
α-tocopherol, μmol/L ^a^	25.5 (0.9)

^a^ Analytes were quantified in fasting plasma samples.

**Table 3:** Pearson’s correlation coefficients between adipose tissue α-TOC concentration and selected anthropometric measurements and blood lipid concentrations.

	Pearson’s *r*	95% CI	*p*-Value
Age	0.25	−0.06, 0.52	0.11
BMI	0.17	−0.14, 0.45	0.28
Fasting lipid concentration			
Total cholesterol	0.36	0.06, 0.60	0.02 ^b^
LDL-C	0.27	−0.07, 0.55	0.11
HDL-C	−0.01	−0.33, 0.32	0.97
Fasting α-TOC/total cholesterol	−0.11	−0.40, 0.20	0.49
Fasting α-TOC/LDL-C	0.13	−0.21, 0.44	0.45
Fasting α-TOC/HDL-C	−0.05	−0.37, 0.28	0.76
Triglycerides (TG)	0.24	−0.07, 0.51	0.12
α-TOC concentration			
in fasting plasma	0.24	−0.07, 0.51	0.14
in postprandial chylomicrons ^a^	0.06	−0.28, 0.38	0.73

^a^ For each participant, the baseline-adjusted area under the curve of the postprandial plasma chylomicron α-TOC concentration over 8 h following the consumption of the α-TOC test meal, an acknowledged marker of VE bioavailability, was calculated [39]. ^b^ Parameters were considered significant at 0.05 level.

**Table 4:** SNPs significantly associated with α-TOC concentration in adipose tissue following univariate analysis.

SNPs	Gene	Alleles	Alternate Allele Frequency (European Population)	Gene Region ^a^	Unstandardized Regression Coefficient ^b^	*p*-Value ^c^	Variant Effect Prediction Score ^d^
Additive model							
rs709158	*PPARG*	A>G	0.35	intron	52.5 ± 14.3	0.0007	0.61
rs1151996	*PPARG*	C>A	0.62	intron	52.6 ± 15.2	0.0013	0.44
rs3211958	*CD36*	A>G	0.45	intron	49.7 ± 16.0	0.0033	0.13
rs2921193	*PPARG*	G>A	0.47	intron	41.8 ± 14.0	0.0048	0.18
rs2575876	*ABCA1*	G>A	0.26	intron	45.3 ± 15.5	0.0057	0.61
rs709150	*PPARG*	C>G	0.39	intron	41.4 ± 14.4	0.0064	0.61
rs4739050	*TTPA*	A>G	0.39	intergenic	−43.7 ± 15.6	0.0078	0.18
rs1152002	*PPARG*	C>T	0.48	intron	39.8 ± 15.5	0.0143	0.61
rs1151998	*PPARG*	A>G	0.47	intron	36.2 ± 14.4	0.0157	0.61
rs7951761	*BUD13*	G>A	0.50	intergenic	39.9 ± 16.3	0.0186	0.13
rs2777788	*ABCA1*	A>G	0.39	intron	36.0 ± 15.8	0.0275	0.61
rs6472073	*TTPA*	C>A	0.48	intergenic	31.6 ± 14.0	0.0295	0.59
rs2297406	*ABCA1*	C>T	0.30	intron	34.5 ± 15.5	0.0312	0.00
rs4823164	*PNPLA3*	C>T	0.46	intergenic	34.2 ± 15.9	0.0372	0.38
rs10891938	*BUD13*	G>A	0.37	intergenic	31.0 ± 15.0	0.0453	0.59
rs2280434	*CYP4F2*	C>A	0.45	3′ UTR	30.2 ± 14.7	0.0468	0.51
rs573126	*BUD13*	A>C	0.32	intergenic	34.0 ± 16.8	0.0491	0.13
rs11216029	*BUD13*	G>T	0.42	intergenic	−33.2 ± 16.4	0.0498	0.13
Dominant Model ^e^							
rs709157	*PPARG*	G>A	0.31	intron	37.1 ± 10.1	0.0007	0.55
rs1561166	*ABCA1*	T>C	0.09	intergenic	56.9 ± 15.6	0.0008	0.13
rs1783225	*BUD13*	T>C	0.11	intergenic	39.2 ± 11.4	0.0013	0.91
rs12686004	*ABCA1*	G>A	0.12	intergenic	42.7 ± 12.5	0.0015	0.59
rs1648364	*BUD13*	T>C	0.13	intergenic	42.5 ± 12.5	0.0016	0.13
rs2275542	*ABCA1*	C>T	0.32	intron	−34.3 ± 10.4	0.0021	0.85
rs4743764	*ABCA1*	T>C	0.42	intron	−34.8 ± 10.9	0.0027	0.61
rs13076933	*PPARG*	T>G	0.26	2 kb upstream	32.9 ± 10.5	0.0032	0.24
rs3773161	*MGLL*	T>C	0.03	intron	49.6 ± 16.2	0.0039	0.69
rs12271395	*BUD13*	A>C	0.17	intergenic	32.7 ± 10.9	0.0044	0.13
rs6778770	*MGLL*	A>G	0.23	intron	29.9 ± 10.7	0.0079	0.51
rs4475472	*TTPA*	T>C	0.08	intergenic	42.7 ± 15.3	0.0079	0.04
rs6008798	*PPARA*	T>C	0.27	intergenic	−29.6 ± 10.7	0.0086	0.61
rs482795	*BUD13*	A>G	0.26	intergenic	32.6 ± 11.9	0.0088	0.13
rs13288647	*PLIN2*	A>G	0.32	intergenic	−29.4 ± 10.8	0.0094	0.00
rs670345	*DGAT2*	G>A	0.07	intergenic	−36.1 ± 13.7	0.0120	0.61
rs608318	*MGLL*	T>G	0.14	intron	−30.4 ± 11.7	0.0131	0.61
rs9289316	*MGLL*	A>G	0.11	intron	43.3 ± 16.7	0.0132	0.61
rs12629751	*PPARG*	C>T	0.09	intron	43.1 ± 16.7	0.0136	0.18
rs3904998	*ABCA1*	T>C	0.21	intron	28.7 ± 11.1	0.0137	0.13
rs135549	*PPARA*	T>C	0.42	intron	−29.7 ± 11.7	0.0153	0.72
rs2622621	*ABCG2*	C>G	0.27	intron	27.4 ± 10.9	0.0157	0.61
rs135552	*PPARA*	T>C	0.27	intron	−27.8 ± 11.1	0.0165	0.33
rs2074303	*TM6SF2*	C>T	0.34	intron	28.5 ± 11.5	0.0180	0.61
rs519000	*BUD13*	C>T	0.16	intergenic	28.4 ± 11.6	0.0184	0.61
rs11716997	*MGLL*	T>G	0.43	intergenic	−31.4 ± 12.8	0.0185	0.36
rs1152001	*PPARG*	A>G	0.21	intron	−26.0 ± 11.2	0.0249	0.61
rs4922131	*LPL*	G>A	0.44	intergenic	28.2 ± 12.1	0.0252	0.55
rs1383194	*NKAIN3*	T>C	0.30	intron	26.8 ± 11.5	0.0252	0.18
rs13270035	*NKAIN3*	A>G	0.16	intergenic	−25.7 ± 11.2	0.0268	0.13
rs4149275	*ABCA1*	A>G	0.18	intron	27.9 ± 12.2	0.0272	0.13
rs3934667	*SF4*	G>T	0.35	2 kb upstream	27.1 ± 11.9	0.0282	0.98
rs929090	*PNPLA3*	A>G	0.47	intergenic	27.0 ± 11.9	0.0288	0.13
rs2886571	*CYP4F2*	T>C	0.25	intron	−25.9 ± 11.4	0.0290	0.18
rs7652615	*MGLL*	T>G	0.16	intron	−26.7 ± 11.9	0.0304	0.52
rs1563325	*NKAIN3*	G>A	0.18	intron	24.8 ± 11.1	0.0307	0.61
rs11204094	*LPL*	A>G	0.43	intergenic	−24.6 ± 11.0	0.0309	0.27
rs1152004	*PPARG*	A>G	0.21	intergenic	25.2 ± 11.4	0.0323	0.61
rs11605293	*BUD13*	C>T	0.09	intergenic	−27.0 ± 12.2	0.0326	0.13
rs17193714	*NKAIN3*	C>T	0.09	intergenic	−31.1 ± 14.0	0.0327	0.13
rs7651814	*MGLL*	C>T	0.16	intron	−26.2 ± 11.9	0.0340	0.98
rs4646437	*CYP3A4*	G>A	0.11	intron	−26.6 ± 12.2	0.0358	0.93
rs2174876	*BUD13*	G>A	0.46	intergenic	−25.3 ± 11.7	0.0373	0.13
rs9919066	*ABCA1*	C>T	0.09	intergenic	−27.9 ± 13.0	0.0380	0.00
rs3219281	*NR1H2*	C>T	0.09	0.5 kb downstream	−33.9 ± 15.8	0.0383	0.39
rs2074296	*TM6SF2*	A>G	0.33	intergenic	25.1 ± 11.8	0.0389	0.67
rs1350057	*NKAIN3*	C>T	0.11	intron	26.8 ± 12.6	0.0396	0.18
rs11215905	*BUD13*	T>C	0.45	intergenic	−24.9 ± 11.7	0.0401	0.00
rs4823153	*PNPLA3*	T>C	0.23	intergenic	−23.4 ± 11.1	0.0414	0.61
rs2740486	*ABCA1*	T>G	0.47	intron	26.5 ± 12.6	0.0421	0.51
rs4425750	*NKAIN3*	C>T	0.11	intron	26.4 ± 12.6	0.0424	0.55
rs479504	*MGLL*	C>A	0.21	intron	25.6 ± 12.3	0.0439	0.76
rs6439099	*MGLL*	T>C	0.08	intergenic	−31.0 ± 14.9	0.0439	0.18
rs3124016	*ABCA1*	G>A	0.26	intergenic	23.4 ± 11.3	0.0453	0.11
rs573713	*BUD13*	A>G	0.13	intergenic	−23.8 ± 11.6	0.0466	0.13
rs11216157	*APOA1*	A>G	0.13	intron	−23.7 ± 11.6	0.0472	0.70
rs10991509	*ABCA1*	A>G	0.28	intergenic	−23.4 ± 11.4	0.0476	0.61
rs11216026	*BUD13*	A>G	0.28	intergenic	−22.6 ± 11.1	0.0484	0.13
rs11215728	*BUD13*	C>T	0.40	intergenic	−23.3 ± 11.5	0.0490	0.61

^a^ Alternate allele frequencies were retrieved from dbSNP (https://www.ncbi.nlm.nih.gov/snp/, accessed on 1 March 2023) using the Allele Frequency Aggregator (ALFA) dataset (pooled allele frequency data from dbSNP and the dbGaP) in the European population (1 March 2023). Coding status: intron, missense, upstream, or untranslated region (UTR). The 3′ UTR is a regulatory DNA region situated at the 3′ end of all protein-coding genes that is transcribed into mRNA but not translated into protein. All SNPs were otherwise intergenic. ^b^ Unstandardized regression coefficients represent the mean change in α-TOC concentration in the adipose tissue (μmol/g protein) for each additional copy of the minor allele under the additive model and in the presence of the minor allele under the dominant model. ^c^ SNPs are ranked by increasing *p*-values. ^d^ Variant Effect Prediction Score was estimated using RegulomeDB for intron, upstream, UTR, or intergenic SNPs (accessed on 9 May 2023). ^e^ For SNPs under the dominant model, participants homozygous for the lesser frequent allele were grouped with heterozygous participants and were compared with participants homozygous for the more frequent allele. Abbreviations: Gene names can be found in Supplementary Table S1.

**Table 5:** Combination of SNPs associated with α-TOC concentration in the adipose tissue following partial least squares regression.

Gene ^a^	SNP	VIP Value ^b^	Regression Coefficient ^c^
*PPARG*	rs709158	1.35	16.8
*ABCA1*	rs1561166	1.34	36.4
*BUD13*	rs1783225	1.29	25.0
*ABCA1*	rs12686004	1.28	27.3
*BUD13*	rs1648364	1.27	27.2
*ABCA1*	rs2275542	1.24	21.9
*ABCA1*	rs4743764	1.20	22.3
*PPARG*	rs13076933	1.20	21.0
*CD36*	rs3211958	1.19	15.9
*MGLL*	rs3773161	1.17	31.7

^a^ Gene names can be found in Supplementary Table S1. ^b^ Variables were ranked according to their variable importance in the projection (VIP) value, which estimates the contribution of each SNP in the projection used in the PLS regression model. Note that 3 out of the 12 SNPs present in the selected model were in LD. Since these SNPs provided redundant information to the model, we kept only the one with the highest VIP in the final selected partial least squares regression model. See (Supplementary Table S5) for the identification of SNPs in LD. ^c^ Regression coefficients are for untransformed variables and represent the mean change in α-TOC concentration in adipose tissue (μmol/g protein) for each additional copy of the minor allele under the additive model and in the presence of the minor allele under the dominant model.

## Data Availability

The data presented in this study are available on request from the corresponding author because this was not stated in the ethics application.

## Supplementary Materials

The following supporting information can be downloaded at: https://www.mdpi.com/article/10.3390/nu16152556/s1, Table S1: Candidate genes selected; Table S2: Characteristics of the partial least squares regression models generated; Table S3: (A) Effect of time and type of meal on adipose tissue α-TOC concentrations. (B) Effect of time within each meal on adipose tissue α-TOC concentrations; Figure S1: Candidate SNP selection flowchart; Table S4: Average relative prediction error following the leave-*k*-out procedure; Figure S2: SNPs stability following the leave-k-out procedure. k participants (*k* = {1,2,3,4}) were randomly removed from the original dataset, thus leaving a training subset; Figure S3: The horizontal axis represents the correlation between the permuted Y’s and the original Y’s; Table S5: SNPs in LD in the final PLS regression model. Figure S4: Retrospective multivariate power calculation was performed for a PLS model that incorporated 10 SNP variables (refer to Table 5, main manuscript) to differentiate participants with low and high adipose tissue α-TOC concentration.
