# Supplementary material for: Genetic Factors Contributing to Interindividual Variability of α-Tocopherol Levels in Subcutaneous Adipose Tissue among Healthy Adult Males
Source: Nutrients. 2024 Aug 3;16(15):2556. doi: 10.3390/nu16152556 (PMC11314220; doi:10.3390/nu16152556)
Supplement: Supplementary file 1 [file nutrients-16-02556-s001.zip › nutrients-3115451-supplementary.pdf]

Genetic factors contributing to interindividual variability in α-tocopherol levels in subcutaneous adipose tissue among healthy adult males

Mark Pretzel Zumaraga, Patrick Borel, Beatrice Gleize, Marion Nowicki, Djaffar Ould-Ali, Jean-François Landrier[1] and Charles Desmarchelier

Supporting Tables, Figures and Methods

Supplemental Table S1. Candidate genes selected.

Gene Name	Gene symbol	References
Genes that play, or are assumed to play, a role in alpha-tocopherol and triglyceride metabolism in adipose tissue		
**ATP binding cassette subfamily A member 1**	*ABCA1*	[1,2]
**ATP binding cassette subfamily B member 1**	*ABCB1*	[3]
**ATP binding cassette subfamily G member 1**	*ABCG1*	[4,5]
**ATP binding cassette subfamily G member 2**	*ABCG2*	[6,7]
**Apolipoprotein A1** [a]	*APOA1*	[8–10]
**Apolipoprotein A4** [a]	*APOA4*	[8–10]
**Apolipoprotein A5** [a]	*APOA5*	[8–10]
**Apolipoprotein C3** [a]	*APOC3*	[8–10]
**Cluster of Differentiation 36**	*CD36*	[11]
**Cytochrome P450 family 3 subfamily A member 4**	*CYP3A4*	[12]
**Diacylglycerol O-acyltransferase 2**	*DGAT2*	[13]
**Low density lipoprotein receptor**	*LDLR*	[11,14]
**Lipase E, hormone sensitive type**	*LIPE*	[15]
**Lipoprotein lipase**	*LPL*	[11,16]
**Monoglyceride lipase**	*MGLL*	[15,17]

**NPC intracellular cholesterol transporter 1**	*NPC1*	[18]
**NPC intracellular cholesterol transporter 2**	*NPC2*	[18]
**Nuclear receptor subfamily 1 group H member 2**	*NR1H2*	[5]
**Nuclear receptor subfamily 1 group H member 3**	*NR1H3*	[11]
**Perilipin 2**	*PLIN2*	[19]
**Phospholipid transfer protein**	*PLTP*	[11,20]
**Patatin-like Phospholipase Domain-containing 2**	*PNPLA2*	[15]
**Patatin like phospholipase domain containing 3**	*PNPLA3*	[15]
**Peroxisome proliferator activated receptor alpha**	*PPARA*	[21]
**Peroxisome proliferator activated receptor gamma**	*PPARG*	[11,22,23]
**Secretion associated Ras related GTPase 1B**	*SAR1B*	[7,24]
**Scavenger receptor class B member 1**	*SCARB1*	[11,25]
**Sterol carrier protein 2**	*SCP2*	[7,26]
**SEC14 like lipid binding 2**	*SEC14L2*	[27,28]
**SEC14 like lipid binding 3**	*SEC14L3*	[27,28]
**SEC14 like lipid binding 4**	*SEC14L4*	[27,28]
**Sterol regulatory element binding transcription factor 2**	*SREBF2*	[7,29]
**Alpha tocopherol transfer protein**	*TTPA*	[11,28]
Genes that have been associated with blood concentrations of alpha tocopherol in genome wide association studies (GWAS)		
**BUD13 homolog[a]**	*BUD13*	[30,31]
**Cytochrome P450 family 4 subfamily F member 2**	*CYP4F2*	[31]
**Sodium/potassium transporting ATPase interacting 3**	*NKAIN3*	[31]

**SURP and G-patch domain containing 1**	*SF4*	[32]
**Transmembrane 6 superfamily member 2**	*TM6SF2*	[32]
**ZPR1 zinc finger**	*ZPR1*	[30,31]

[a]These genes belong to *APOA1*/*C3*/*A4*/*A5* gene cluster.

Supplemental Table S2. Characteristics of the partial least squares regression models generated.[a]

**Number of predictors**	**R^2**	**Adjusted R^2**	**R^2 after 100 permutations[b]**	**R^2 after cross-validation[b]**	**Cross-validation-ANOVA *p*-value[c]**
78 (77 SNPs and fasting cholesterol)	0.80	1.22	0.45	0.71	2.55×10^{-11}
38	0.75	-2.4	0.32	0.66	6.29×10^{-10}
24	0.71	0.31	0.25	0.64	2.94×10^{-9}
22	0.71	0.38	0.22	0.63	3.58×10^{-9}
17	0.63	0.37	0.18	0.63	2.17×10^{-7}
16	0.62	0.37	0.16	0.54	3.32×10^{-7}
15	0.64	0.43	0.17	0.56	1.10×10^{-7}
14	0.65	0.46	0.16	0.58	5.17×10^{-8}
**12**	**0.67**	**0.53**	**0.17**	**0.60**	**1.61×10^{-8}**
10	0.60	0.47	0.17	0.52	5.65×10^{-7}
9	0.61	0.50	0.13	0.54	2.78×10^{-7}
8	0.59	0.49	0.11	0.52	5.98×10^{-7}
7	0.57	0.48	0.11	0.51	1.07×10^{-6}
5	0.49	0.42	0.07	0.43	1.93×10^{-5}

[a] Different partial least squares regression models were built using increasing VIP threshold values. The selected model is highlighted in bold font following the selection criteria previously detailed in the Methods section under the subheading **Statistical analysis**. All models had one component.

[b]See **Supplemental Figure S3** for further explanation of the procedure.

[c]See [33].

Supplemental Table S3. (A) Effect of time and type of meal on adipose tissue α-TOC concentrations. **(B)** Effect of time within each meal on adipose tissue α-TOC concentrations.

(A)

**Parameters**[a]	**Numerator *df***	**Denominator *df***	**F**	**Sig.**[b]
Intercept	1	39.576	104.125	0.000
Time (Fasting vs 8 hr)	1	30.662	0.007	0.935
Type of Meal (Control vs α-TOC vs Tomato Puree)	2	39.570	0.311	0.734
Time * Type of Meal	2	37.989	1.128	0.334

(B)

**Type of Meal**	**Paired Differences**[c]					**t**	***df***	**Sig.**[d]
				**95% CI**				
	**Mean**	**SD**	**SEM**	**Lower**	**Upper**			
Control Meal	-44.141	251.299	43.098	-131.824	43.542	-1.024	33	0.313
α-TOC Meal	19.034	121.387	21.458	-24.731	62.798	0.887	31	0.382
Tomato Puree Meal	20.416	137.148	23.182	-26.696	67.528	0.881	34	0.385

[a]Unstructured mixed model. Adipose tissue α-TOC concentrations measured at fast and 8 h after consumption of the 3 test meals was analyzed with linear mixed models, using a full factorial design with Type of Meal (control, α-TOC and tomato puree) and Time (fasting and 8 h post-meal) as fixed within-subject variables and participant as the random variable. Of the 5 linear mixed models tested, the unstructured model was selected based on Akaike's Information Criterion [34]. [b] Parameters were considered significant at 0.05 level. [c] The paired differences of adipose tissue α-TOC concentrations between fasting and after 8 h consumption of test meals in 42 participants are displayed. [d] Two-way significance test was performed. A *p*-value less than 0.05 comparing adipose tissue α-TOC concentration before and after intake of each test meal was considered significant.

Supplemental Figure S1. Candidate SNP selection flowchart.

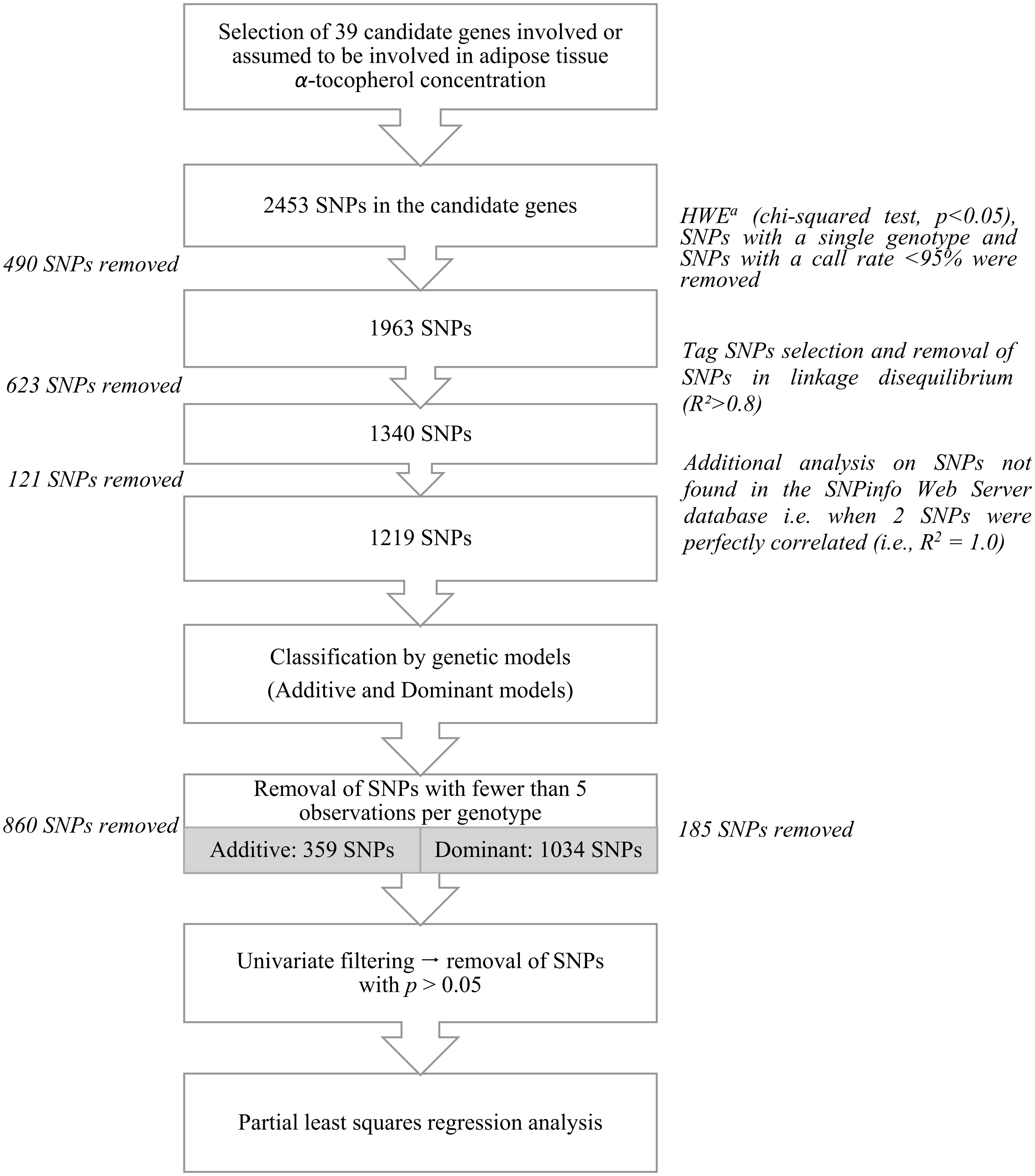

[a]Deviations from Hardy-Weinberg equilibrium (HWE) can indicate inbreeding, population stratification, and genotyping errors.

Supplemental Information: additional validations of the partial least squares (PLS) regression model.

1) *Leave*-k-*out* cross-validation

The leave *k*-out validation procedure was based on Steyerberg *et al.* [35]. Briefly, we challenged our PLS regression model by randomly taking out *k* participants (k={1,2,3,4}) from the original dataset, thus leaving a training dataset. The *k* participants taken out were then reintroduced into this training set to assess whether the models built without these *k* participants were able to predict their adipose tissue *α*-TOC concentration accurately. This test was performed as many times so that each participant was taken out once (*i.e.* 42 times for k=1, 21 times for k=2, 14 times for k=3 and 10 times for k=4).

The *% error* between the predicted and the measured adipose tissue *α*-TOC concentration for each *k* are shown in **Supplemental Table S4**. The percentage of error remained relatively stable, even when up to 4 participants were left out of the model, suggesting that the PLS regression model was relatively robust.

Supplemental Table S4. Average relative prediction error following the leave-*k*-out procedure.

	**Number of participants left out**				
	0	1	2	3	4
% error	35.4	39.1	38.7	38.7	39.5

2) *Regression coefficient stability testing following the leave-*k*-out procedure*

We checked that the regression coefficients of the 10 SNPs from the selected model (**Table 5**) remained unchanged (*p*>0.05; ANOVA) following the leave-*k*-out procedure described above. **Supplemental Figure S2** shows good stability of the regression coefficients with this validation.

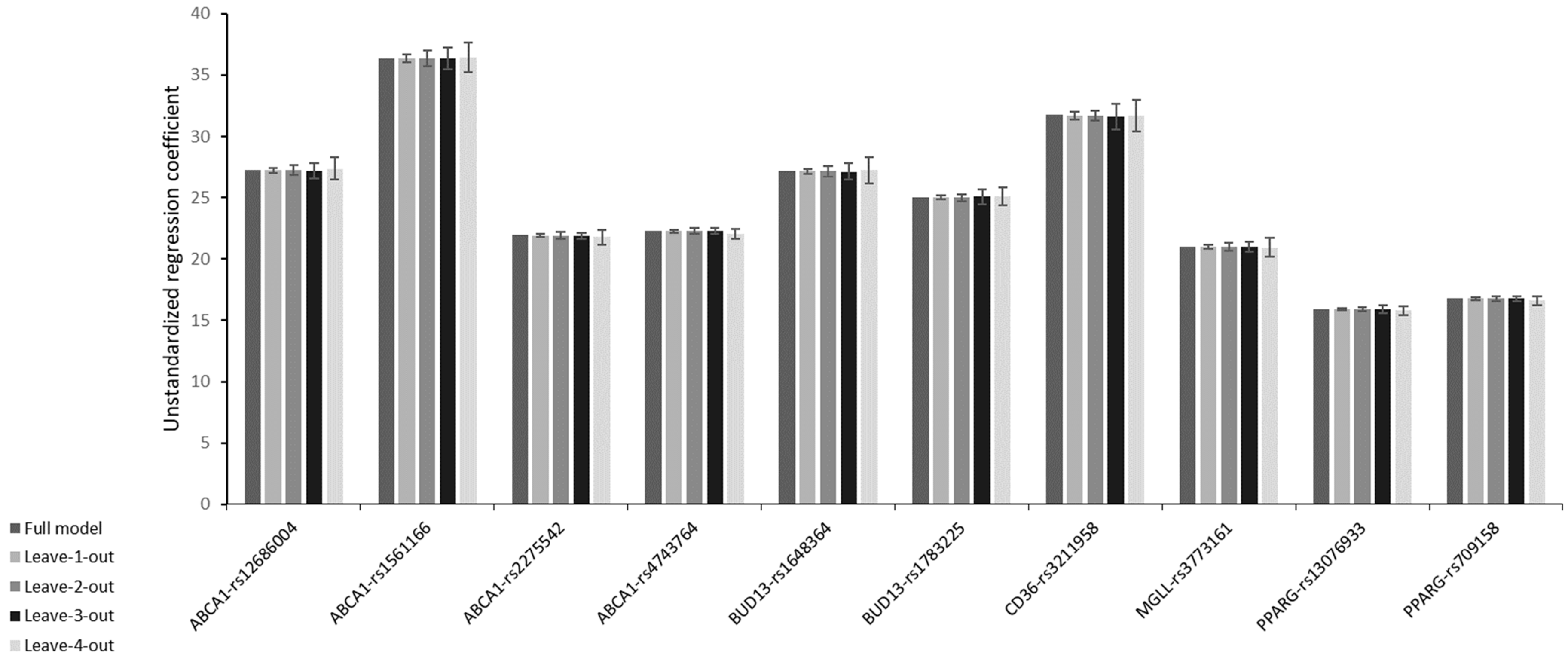

Supplemental Figure S2: SNPs stability following the leave-*k*-out procedure. *k* participants (k={1,2,3,4}) were randomly removed from the original dataset, thus leaving a training subset. These participants were then reintroduced in the training subset to assess the regression coefficients of the 10 SNPs from the selected model. This test was performed as many times so that each participant was taken out once. One-way ANOVA

performed for each gene showed no significant differences between the 4 training subsets generated by the procedure. Gene names are found in **Supplemental Table S1**.

3) R^2 *and adjusted* R^2 *of the selected model after 100 permutations.*

This procedure 1) assesses the risk that the PLS regression model is spurious, *i.e.* the model fits the current data set well but does not predict Y well for new observations, and 2) tests for over-fitting. For over-fitting, the accuracy of fit (R^2 and R^2 after cross-validation) of the original model was compared with the accuracy of fit of 100 models based on data where the order of the Y matrix for the participants (adipose tissue α-TOC concentration) was randomly permuted, while the X matrix (the genotypes at the selected SNPs) was kept intact. Thus, a robust model (where the fit between X and Y is high) should be unable to predict the permuted Y variables with the intact X variables. **Supplemental Figure S3** shows the results of these permutations for the selected PLS regression model.

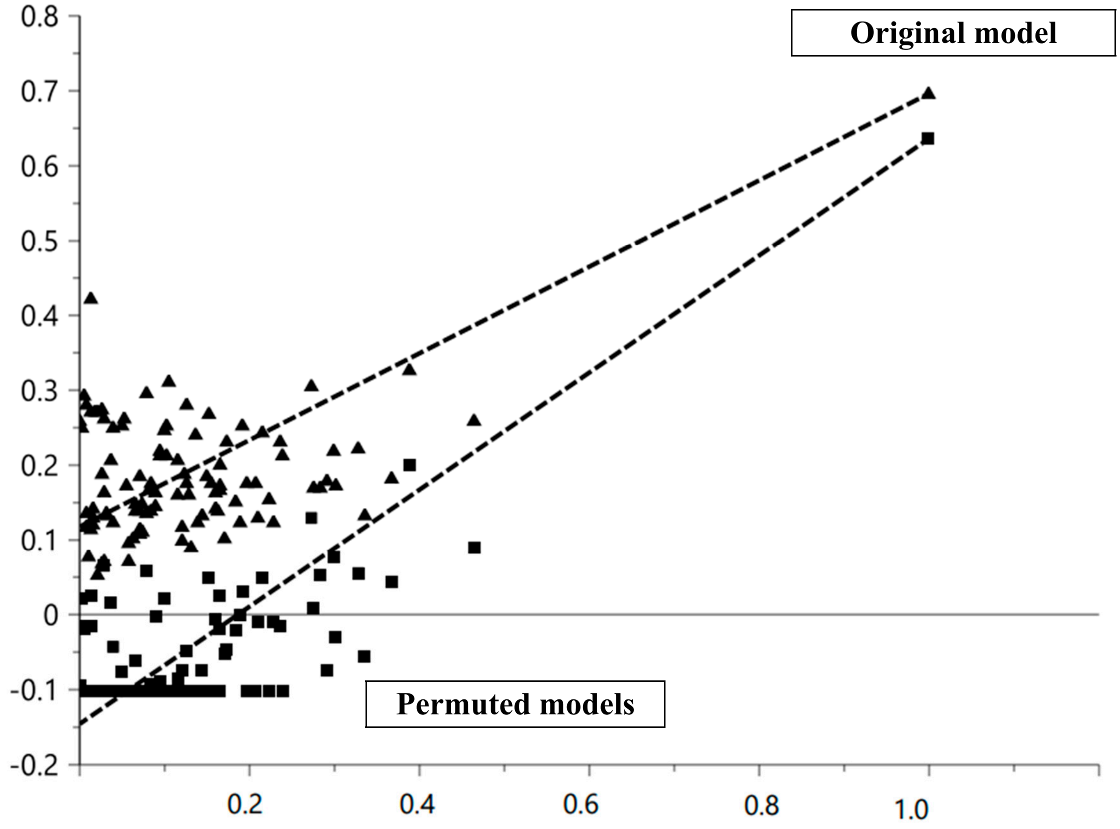

Supplemental Figure S3. The horizontal axis represents the correlation between the permuted Y's and the original Y's. The vertical axis represents the R^2 (dashed line and black triangles)

and R^2 after cross-validation (dashed line and squares) values obtained in the permuted models. Values of the original model are on the far right (at correlation = 1), values of the 100 *Y*-permuted models are further to the left. The average R^2 after 100 permutations was 0.23. This strongly supports the conclusion that the ability of the original, non-permuted model, to predict the adipose tissue α-TOC concentration is not due to chance.

Pairwise LD test: Identification of LD SNPs in the final PLS model

The selected PLS regression model contained 12 SNPs (**Supplemental Table S2**). Three were in LD according to an online calculator tool for pairwise LD (available at https://www.ensembl.org/Homo_sapiens/Tools/LD , population: European CEU, queried in 28 March 2024)**.** Since these SNPs provided redundant information in the model, we kept the one which had the highest VIP, presented in the right column, leaving 10 SNPs in the final selected PLS regression model (**Table 5**).

Supplemental Table S5. SNPs in LD in the final PLS regression model.[a]

SNP not retained in the final PLS regression model	VIP[b]	SNP retained in the final PLS regression model	VIP[b]	LD r^2[c]
rs709157	1.351	rs709158	1.353	0.87
rs1151996	1.293	rs709158	1.353	0.91

[a]Gene names can be found in Supplemental Table S1.

[b] VIP: Variable Importance in Projection

[c]Pairwise LD r^2 results were generated from population 1000GENOMES:phase_3:CEU (accessible at https://www.ensembl.org/Homo_sapiens/Tools/LD)

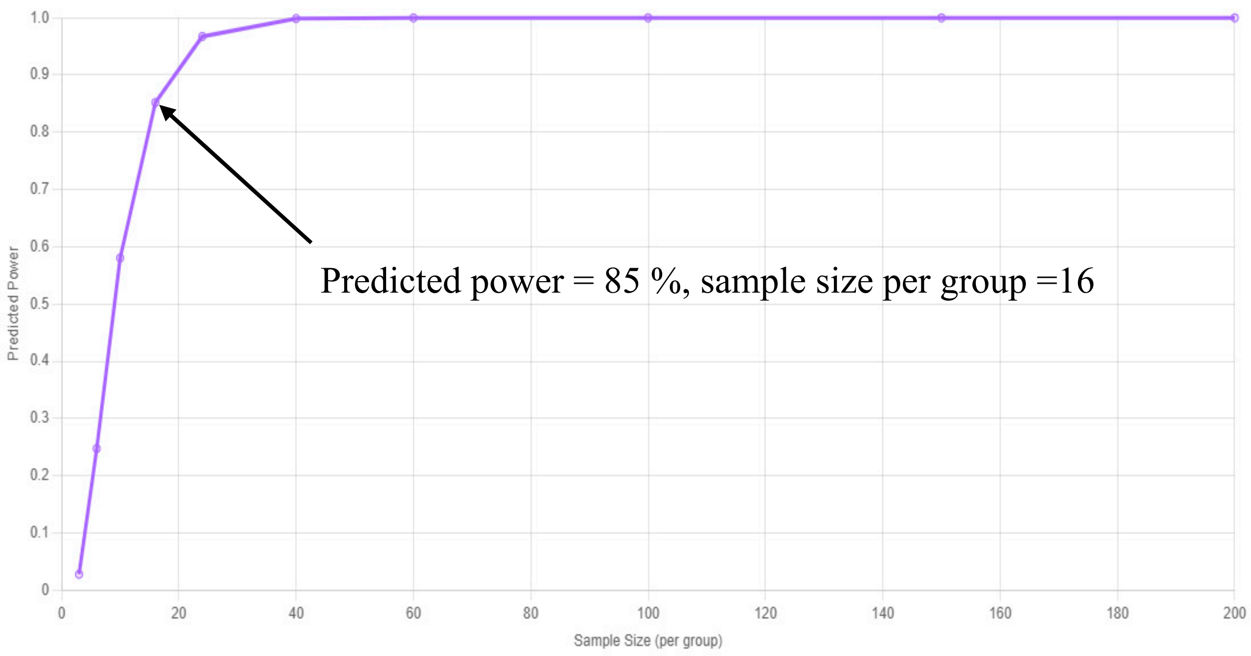

Supplemental Figure S4. Retrospective multivariate power calculation was performed for a PLS model that incorporated 10 SNP variables (refer to **Table 5**, main manuscript) to differentiate participants with low and high adipose tissue α-TOC concentration. With an FDR-adjusted p-value of 0.001, the predicted multivariate power of 85 % (vertical axis) was calculated for a sample size of 16 per group (horizontal axis), confirming that the sample size selected for this study was sufficient. Image obtained from MetaboAnalyst 6.0 website, accessible at https://new.metaboanalyst.ca.

References:

1. Traber, M.G. Mechanisms for the Prevention of Vitamin E Excess. *J Lipid Res* **2013**, *54*, 2295–2306, doi:10.1194/jlr.R032946.
2. Oram, J.F.; Vaughan, A.M.; Stocker, R. ATP-Binding Cassette Transporter A1 Mediates Cellular Secretion of Alpha-Tocopherol. *J Biol Chem* **2001**, *276*, 39898–39902, doi:10.1074/jbc.M106984200.
3. Mostafa, A.M.; Hamdy, N.M.; Abdel-Rahman, S.Z.; El-Mesallamy, H.O. Effect of Vildagliptin and Pravastatin Combination on Cholesterol Efflux in Adipocytes. *IUBMB Life* **2016**, *68*, 535–543, doi:10.1002/iub.1510.
4. Cavelier, C.; Lorenzi, I.; Rohrer, L.; von Eckardstein, A. Lipid Efflux by the ATP-Binding Cassette Transporters ABCA1 and ABCG1. *Biochim Biophys Acta* **2006**, *1761*, 655–666, doi:10.1016/j.bbalip.2006.04.012.
5. Rode, S.; Rubic, T.; Lorenz, R.L. Alpha-Tocopherol Disturbs Macrophage LXRalpha Regulation of ABCA1/G1 and Cholesterol Handling. *Biochem Biophys Res Commun* **2008**, *369*, 868–872, doi:10.1016/j.bbrc.2008.02.132.
6. Sarkadi, B.; Ozvegy-Laczka, C.; Német, K.; Váradi, A. ABCG2 -- a Transporter for All Seasons. *FEBS Lett* **2004**, *567*, 116–120, doi:10.1016/j.febslet.2004.03.123.
7. Borel, P.; Desmarchelier, C.; Nowicki, M.; Bott, R.; Tourniaire, F. Can Genetic Variability in α-Tocopherol Bioavailability Explain the Heterogeneous Response to α-Tocopherol Supplements? *Antioxid Redox Signal* **2015**, *22*, 669–678, doi:10.1089/ars.2014.6144.
8. Zhu, Q.; Weng, J.; Shen, M.; Fish, J.; Shen, Z.; Coschigano, K.T.; Davidson, W.S.; Tso, P.; Shi, H.; Lo, C.C. Apolipoprotein A-IV Enhances Fatty Acid Uptake by Adipose Tissues of Male Mice via Sympathetic Activation. *Endocrinology* **2020**, *161*, bqaa042, doi:10.1210/endocr/bqaa042.
9. Frances, L.; Tavernier, G.; Viguerie, N. Adipose-Derived Lipid-Binding Proteins: The Good, the Bad and the Metabolic Diseases. *Int J Mol Sci* **2021**, *22*, 10460, doi:10.3390/ijms221910460.
10. Huebbe, P.; Lange, J.; Lietz, G.; Rimbach, G. Dietary Beta-carotene and Lutein Metabolism Is Modulated by the APOE Genotype. *Biofactors* **2016**, *42*, 388–396.
11. Zingg, J.-M. Vitamin E: Regulatory Role on Signal Transduction. *IUBMB Life* **2019**, *71*, 456–478, doi:10.1002/iub.1986.
12. Sontag, T.J.; Parker, R.S. Cytochrome P450 Omega-Hydroxylase Pathway of Tocopherol Catabolism. Novel Mechanism of Regulation of Vitamin E Status. *J Biol Chem* **2002**, *277*, 25290–25296, doi:10.1074/jbc.M201466200.
13. Hou, X.G.; Moser, S.; Sarr, M.G.; Thompson, G.B.; Que, F.G.; Jensen, M.D. Visceral and Subcutaneous Adipose Tissue Diacylglycerol Acyltransferase Activity in Humans. *Obesity (Silver Spring)* **2009**, *17*, 1129–1134, doi:10.1038/oby.2008.659.
14. Meydani, M.; Kwan, P.; Band, M.; Knight, A.; Guo, W.; Goutis, J.; Ordovas, J. Long-Term Vitamin E Supplementation Reduces Atherosclerosis and Mortality in Ldlr-/- Mice, but Not When Fed Western Style Diet. *Atherosclerosis* **2014**, *233*, 196–205, doi:10.1016/j.atherosclerosis.2013.12.006.
15. Santucci, P.; Canaan, S. Lipid Droplets Breakdown: Adipose Triglyceride Lipase Leads the Way. *Curr Protein Pept Sci* **2018**, *19*, 1131–1133, doi:10.2174/1389203719666180809143000.
16. Weinstock, P.H.; Levak-Frank, S.; Hudgins, L.C.; Radner, H.; Friedman, J.M.; Zechner, R.; Breslow, J.L. Lipoprotein Lipase Controls Fatty Acid Entry into Adipose Tissue, but Fat Mass Is Preserved by Endogenous Synthesis in Mice Deficient in Adipose Tissue

Lipoprotein Lipase. *Proc Natl Acad Sci U S A* **1997**, *94*, 10261–10266, doi:10.1073/pnas.94.19.10261.
17. Vaughan, M.; Berger, J.E.; Steinberg, D. Hormone-Sensitive Lipase and Monoglyceride Lipase Activities in Adipose Tissue. *J Biol Chem* **1964**, *239*, 401–409, doi:10.1016/S0021-9258(18)51692-6.
18. Xu, M.; Liu, K.; Swaroop, M.; Porter, F.D.; Sidhu, R.; Firnkes, S.; Ory, D.S.; Marugan, J.J.; Xiao, J.; Southall, N.; et al. δ-Tocopherol Reduces Lipid Accumulation in Niemann-Pick Type C1 and Wolman Cholesterol Storage Disorders. *J Biol Chem* **2012**, *287*, 39349–39360, doi:10.1074/jbc.M112.357707.
19. Schmölz, L.; Schubert, M.; Kirschner, J.; Kluge, S.; Galli, F.; Birringer, M.; Wallert, M.; Lorkowski, S. Long-Chain Metabolites of Vitamin E: Interference with Lipotoxicity via Lipid Droplet Associated Protein PLIN2. *Biochim Biophys Acta Mol Cell Biol Lipids* **2018**, *1863*, 919–927, doi:10.1016/j.bbalip.2018.05.002.
20. Lemaire-Ewing, S.; Desrumaux, C.; Néel, D.; Lagrost, L. Vitamin E Transport, Membrane Incorporation and Cell Metabolism: Is Alpha-Tocopherol in Lipid Rafts an Oar in the Lifeboat? *Mol Nutr Food Res* **2010**, *54*, 631–640, doi:10.1002/mnfr.200900445.
21. Juretić, N.; Sepúlveda, R.; D'Espessailles, A.; Vera, D.B.; Cadagan, C.; de Miguel, M.; González-Mañán, D.; Tapia, G. Dietary Alpha- and Gamma-Tocopherol (1:5 Ratio) Supplementation Attenuates Adipose Tissue Expansion, Hepatic Steatosis, and Expression of Inflammatory Markers in a High-Fat-Diet-Fed Murine Model. *Nutrition* **2021**, *85*, 111139, doi:10.1016/j.nut.2021.111139.
22. Landrier, J.-F.; Gouranton, E.; El Yazidi, C.; Malezet, C.; Balaguer, P.; Borel, P.; Amiot, M.-J. Adiponectin Expression Is Induced by Vitamin E via a Peroxisome Proliferator-Activated Receptor Gamma-Dependent Mechanism. *Endocrinology* **2009**, *150*, 5318–5325, doi:10.1210/en.2009-0506.
23. Willems, S.; Gellrich, L.; Chaikuad, A.; Kluge, S.; Werz, O.; Heering, J.; Knapp, S.; Lorkowski, S.; Schubert-Zsilavecz, M.; Merk, D. Endogenous Vitamin E Metabolites Mediate Allosteric PPARγ Activation with Unprecedented Co-Regulatory Interactions. *Cell Chem Biol* **2021**, *28*, 1489-1500.e8, doi:10.1016/j.chembiol.2021.04.019.
24. Marcil, V.; Seidman, E.; Sinnett, D.; Sanchez, R.; Spahis, S.; Sané, A.; Levy, E. Tissue Distribution and Regulation of the Small Sar1b GTPase in Mice. *Cell Physiol Biochem* **2014**, *33*, 1815–1826, doi:10.1159/000362960.
25. Yvan-Charvet, L.; Bobard, A.; Bossard, P.; Massiéra, F.; Rousset, X.; Ailhaud, G.; Teboul, M.; Ferré, P.; Dagher, G.; Quignard-Boulangé, A. In Vivo Evidence for a Role of Adipose Tissue SR-BI in the Nutritional and Hormonal Regulation of Adiposity and Cholesterol Homeostasis. *Arterioscler Thromb Vasc Biol* **2007**, *27*, 1340–1345, doi:10.1161/ATVBAHA.106.136382.
26. Gianotti, A.R.; Ferreyra, R.G.; Ermácora, M.R. Binding Properties of Sterol Carrier Protein 2 (SCP2) Characterized Using Laurdan. *Biochim Biophys Acta Proteins Proteom* **2018**, *1866*, 1143–1152, doi:10.1016/j.bbapap.2018.08.007.
27. Saito, K.; Tautz, L.; Mustelin, T. The Lipid-Binding SEC14 Domain. *Biochim Biophys Acta* **2007**, *1771*, 719–726, doi:10.1016/j.bbalip.2007.02.010.
28. Zimmer, S.; Stocker, A.; Sarbolouki, M.N.; Spycher, S.E.; Sassoon, J.; Azzi, A. A Novel Human Tocopherol-Associated Protein: Cloning, in Vitro Expression, and Characterization. *J Biol Chem* **2000**, *275*, 25672–25680, doi:10.1074/jbc.M000851200.
29. He, W.; Xu, Y.; Ren, X.; Xiang, D.; Lei, K.; Zhang, C.; Liu, D. Vitamin E Ameliorates Lipid Metabolism in Mice with Nonalcoholic Fatty Liver Disease via Nrf2/CES1 Signaling Pathway. *Dig Dis Sci* **2019**, *64*, 3182–3191, doi:10.1007/s10620-019-05657-9.

30. Major, J.M.; Yu, K.; Weinstein, S.J.; Berndt, S.I.; Hyland, P.L.; Yeager, M.; Chanock, S.; Albanes, D. Genetic Variants Reflecting Higher Vitamin e Status in Men Are Associated with Reduced Risk of Prostate Cancer. *J Nutr* **2014**, *144*, 729–733, doi:10.3945/jn.113.189928.
31. Major, J.M.; Yu, K.; Chung, C.C.; Weinstein, S.J.; Yeager, M.; Wheeler, W.; Snyder, K.; Wright, M.E.; Virtamo, J.; Chanock, S.; et al. Genome-Wide Association Study Identifies Three Common Variants Associated with Serologic Response to Vitamin E Supplementation in Men. *J Nutr* **2012**, *142*, 866–871, doi:10.3945/jn.111.156349.
32. Wood, A.R.; Perry, J.R.B.; Tanaka, T.; Hernandez, D.G.; Zheng, H.-F.; Melzer, D.; Gibbs, J.R.; Nalls, M.A.; Weedon, M.N.; Spector, T.D.; et al. Imputation of Variants from the 1000 Genomes Project Modestly Improves Known Associations and Can Identify Low-Frequency Variant-Phenotype Associations Undetected by HapMap Based Imputation. *PLoS One* **2013**, *8*, e64343, doi:10.1371/journal.pone.0064343.
33. Eriksson, L.; Trygg, J.; Wold, S. CV-ANOVA for Significance Testing of PLS and OPLS® Models. *J Chemom* **2008**, *22*, 594–600.
34. Bozdogan, H. Model Selection and Akaike's Information Criterion (AIC): The General Theory and Its Analytical Extensions. *Psychometrika* **1987**, *52*, 345–370, doi:10.1007/BF02294361.
35. Steyerberg, E.W.; Harrell Jr, F.E.; Borsboom, G.J.J.M.; Eijkemans, M.J.C.; Vergouwe, Y.; Habbema, J.D.F. Internal Validation of Predictive Models: Efficiency of Some Procedures for Logistic Regression Analysis. *J Clin Epidemiol* **2001**, *54*, 774–781.
